# Pan-Cancer Analysis of Microfibrillar-Associated Protein 2 (MFAP2) Based on Bioinformatics and qPCR Verification

**DOI:** 10.1155/2022/8423173

**Published:** 2022-02-15

**Authors:** Zhilei Qiu, Miao Xin, Chenyang Wang, Yueli Zhu, Qingnuan Kong, Zhijun Liu

**Affiliations:** ^1^Department of Urology, Qingdao Municipal Hospital, Qingdao, Shandong 266071, China; ^2^Department of Anesthesiology, Qingdao Municipal Hospital, Qingdao, Shandong 266071, China; ^3^Qingdao University, Qingdao, Shandong 266071, China; ^4^Department of Radiology, Qingdao Municipal Hospital, Qingdao, Shandong 266071, China; ^5^Department of Pathology, Qingdao Municipal Hospital, Qingdao, Shandong 266071, China

## Abstract

MFAP2 has been reported to play an oncogenic role in several types of human cancers. However, the expression profile of MFAP2 in various cancers and its impact on prognosis and immune infiltration remain unclear. In this study, the mRNA expression and protein expression of MFAP2 in normal tissues, tumor cell lines, and 33 malignant tumor tissues were analyzed comprehensively using Genotype-Tissue Expression (GTEx), Cancer Cell Line Encyclopedia (CCLE), and The Cancer Genome Atlas (TCGA), Oncomine and UALCAN databases, and the expression of MFAP2 in different grades and stages of cancers was assessed using Gene Expression Profiling Interactive Analysis 2 (GEPIA2) and Tumor and Immune System Interaction Database (TISIDB). In general, MFAP2 showed distinct expression in most tumor and normal tissues, closely associated with higher tumor grade, higher tumor stage, and poor survival in multiple cancers. A search of the UALCAN database and the cBioPortal database revealed that this difference in mRNA level expression could be partly attributed to abnormal DNA methylation and mutations at the genomic level. In addition, MFAP2 expression was also associated with tumor mutation burden, microsatellite instability, and neoantigens in different cancer types. More importantly, the TIMER and TISIDB databases also showed that MFAP2 levels were significantly correlated with immune infiltration abundance and immune-related gene markers, as well as ESTIMATE scores. By qPCR, MFAP2 expression was validated in four kinds of tumor tissue samples. The present study combined several databases and performed a pan-cancer analysis of the expression profile, methylation, and mutation for MFAP2 and its implications for prognosis and immune infiltration, suggesting that MFAP2 could contribute to malignant properties of many tumors. MFAP2 may be an important biomarker with prognostic value and has the potential to be a target for tumor immunotherapy.

## 1. Introduction

Immunotherapy is considered to be a promising treatment for cancers [1]. However, due to the heterogeneity of tumors, only 10–20% of the population can benefit from current immunotherapy [2, 3]. For example, anti-CTLA4 has poor clinical efficacy in gastric cancer [4]. Anti-PD-1 and anti-PD-L1 have shown partial response in progressive colorectal cancer [5]. With the development of high-throughput sequencing technologies, abundant data are available to the public, such as TCGA database containing transcriptome data for 33 tumors. It is possible and necessary to perform pan-cancer analyses of genes and to assess their correlation with clinical prognosis and immune infiltration [6]. New biomarkers are urgently needed to predict prognosis and to find new immune-related therapeutic targets.

MFAP2 consists of a 183-amino-acid protein with 2 domains [7]. MFAP2 exists in two forms. One is extracellular MFAP2, which is a protein binding to fibrin, collagen VI, tropoelastin, deproteinized, and biglycan14. The other is intracellular protein, which is a functional protein upregulating downstream genes related to cell adhesion, movement, and matrix remodeling. In the last decade, aberrant expression of MFAP2 was found in various malignant tumors. MFAP2 is overexpressed in melanoma with its capacity of manipulating EMT-related proteins and Wnt/*β*-catenin pathway to enhance melanoma invasion and migration ability [8]. Upregulation of MFAP2 in gastric carcinoma has been found, in which MFAP2 accelerates cancer cell migration via MFAP2/integrin *α*5*β*1/FAK/ERK pathway [9, 10]. The ability of MFAP2 in activating TGF-*β*/SMAD2/3 pathway in gastric carcinoma has also been reported, and this activation accelerates the transformation of gastric carcinoma from an epithelial cell phenotype to a mesenchymal phenotype [11]. Previous studies have pointed out that MFAP2 possesses a hyperaffinity to TGF-*β* superfamily member in adipose tissue [12–14].

However, there are no pan-cancer studies on the relationship between MFAP2 and various cancers. Here, we retrieved multiple databases, GTEx, CCLE, Oncomine, TCGA, UALCAN, GEPIA2, and TISIDB, to analyze MFAP2 expression in pan-cancer and its relationship with prognosis. In addition, we explored the relationship between MFAP2 expression and gene mutations, promoter methylation, tumor neoantigens, tumor mutation burden (TMB), microsatellite instability (MSI), mismatch repair (MMR) genes, and immune infiltration. Our results suggested that aberrant expression of MFAP2 was associated with its altered promoter methylation, affected immune infiltration in the tumor microenvironment, and also acted as a prognostic risk factor for a variety of cancers. This study was expected to provide a theoretical basis for gaining insight into the role of MFAP2 in tumor immunotherapy.

## 2. Materials and Methods

### 2.1. Gene Expression Analysis

We downloaded the normalized pan-cancer datasets TCGA and GTEx from the UCSC database, extracted the expression data of MFAP2 gene in each sample, and further transformed each expression value as log2(x+1). The MFAP2 expression in 33 cancers was obtained. In addition, data of each tumor cell line were also downloaded from the CCLE database and analyzed the expression levels of MFAP2 in 21 tumor cell lines. Data analysis was performed using RStudio version 1.1.456 (RStudio Inc., USA) and the *R* package ggpubr. Moreover, the expression levels of MFAP2 gene in different cancers were identified in the Oncomine database [15], with the thresholds of *p*-value = 0.05, fold change = 2, and gene rank = ALL. MFAP2 protein expression was investigated in UALCAN database, providing us a platform for protein expression analysis of Clinical Proteomics Cancer Analysis Alliance dataset [16]. Finally, we explored MFAP2 expression in different pathological stages and grades of TCGA tumors via GEPIA2 and TISIDB databases.

### 2.2. Prognostic Analysis

We first analyzed the relationship between MFAP2 expression in the 33 tumors and overall survival (OS) using TCGA data and visualized it with forest plots using univariate Cox regression analysis. Kaplan–Meier curves were further plotted to show the prognostic significance of MFAP2. Considering the possibility of non-tumor-related deaths during follow-up, we analyzed the relationship between MFAP2 expression and disease-specific survival (DSS), disease-free interval (DFI), and progression-free interval (PFI) in the 33 TCGA tumors.

### 2.3. Genetic Changes Analysis

On the website cBioPortal [17], we selected “TCGA Pan Cancer Atlas Studies” datasets. The mutations and copy number of MFAP2 were investigated, and MFAP2 mutation sites were displayed in schematic and 3D structure maps.

The TMB and MSI scores of all samples were determined from the somatic mutation data downloaded from TCGA, and the correlation between MFAP2 expression and TMB and MSI was assessed using Spearman's rank correlation coefficient. The number of tumor neoantigens in each tumor sample was counted and its relationship with MFAP2 expression was analyzed. Moreover, TCGA expression profile data were used to analyze the expression of MMR genes, including MutL homologous gene (MLH1), MutS homologous gene (MSH2), MSH6, increased separation after meiosis (PMS2), and epithelial cell adhesion molecule (EPCAM) in different tumors. The correlation between MFAP2 and MMR genes was visualized in a heat map using the Reshape2 and *R* ColorBrewer packages.

### 2.4. DNA Methylation Analysis

The UALCAN database was used to show the methylation levels of MFAP2 in different tumors and corresponding normal tissues. In addition, we analyzed the correlation between MFAP2 expression and the expression of the four methyltransferases, including DNMT1, DNMT2, DNMT3A, and DNMT3B.

### 2.5. Immune Infiltration Analysis

We used TISIDB database, and TIMER, microenvironment cell populations (MCP)-counter and XCELL algorithms to explore the relationship between MFAP2 expression and immune infiltration in all TCGA tumors. We analyzed the stromal, immune, and ESTIMATE scores of each tumor sample using the ESTIMATE package and visualized the relationship between these scores and MFAP2 expression using scatter plots. In addition, we investigated the correlation of MFAP2 expression with monocyte and macrophage biomarkers using the TIMER database. Furthermore, we performed Spearman correlation analysis of MFAP2 and immune-related genes, including immunoinhibitors, immunostimulators, major histocompatibility complex (MHC) genes, chemokines, chemokine receptors, and immune checkpoints molecules. All data obtained were finally visualized in heat maps or scatter plots.

### 2.6. Tumor Tissue Collection

BLCA, BRCA, HNSC, and KICH tissues and normal tissues were collected from 5 patients, respectively. They were stored immediately in liquid nitrogen and kept at -80 °C. The study was approved by the Ethics Committee of the Qingdao Municipal Hospital of Shandong province and conducted following the Declaration of Helsinki.

### 2.7. Quantitative Real-Time Polymerase Chain Reaction (qPCR)

Total RNA was extracted from tissues using TRIzol reagent (Invitrogen, Thermo Fisher Scientific, Shanghai, China) and reverse-transcribed into cDNA using cDNA Reverse Transcription Kit (Applied Biosystems, Foster City, CA, USA). qPCR was performed using SYBR Premix Ex Taq II kit (RR820 A, TaKaRa, Dalian, China), with GAPDH as an internal reference. The relative expression of MFAP2 was calculated using the 2^−ΔΔCt^ method. The primers used are listed in Table 1.

### 2.8. Statistical Analysis

The data were analyzed using *R* 4.0.2 software and GraphPad 9.0.0 and expressed as mean ± SD. Differences were analyzed by *t*-test, with *p* values less than 0.05 considered statistically significant.

## 3. Results

### 3.1. MFAP2 Expression across Cancers

**Figure 1:**
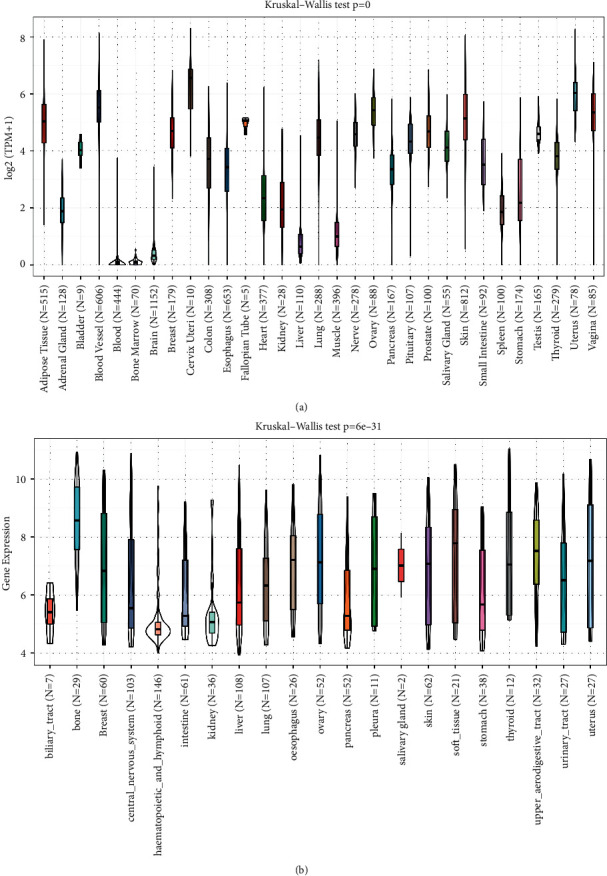
MFAP2 basal level. (a) MFAP2 expression in normal tissues obtained from GTEx database. (b) MFAP2 expression in tumor cell lines obtained from CCLE database.

We first examined the expression levels of MFAP2 in normal tissues via the GTEx dataset. As shown in Figure 1(a), MFAP2 expression levels were the highest in cervix uteri tissues and the lowest in blood and bone marrow tissues. Also, the basal levels of MFAP2 expression in various tumor cell lines were assessed. As shown in Figure 1(b), MFAP2 was expressed at the highest level in bone cell lines and the lowest level in haematopoietic and lymphoid cell lines.

**Figure 2:**
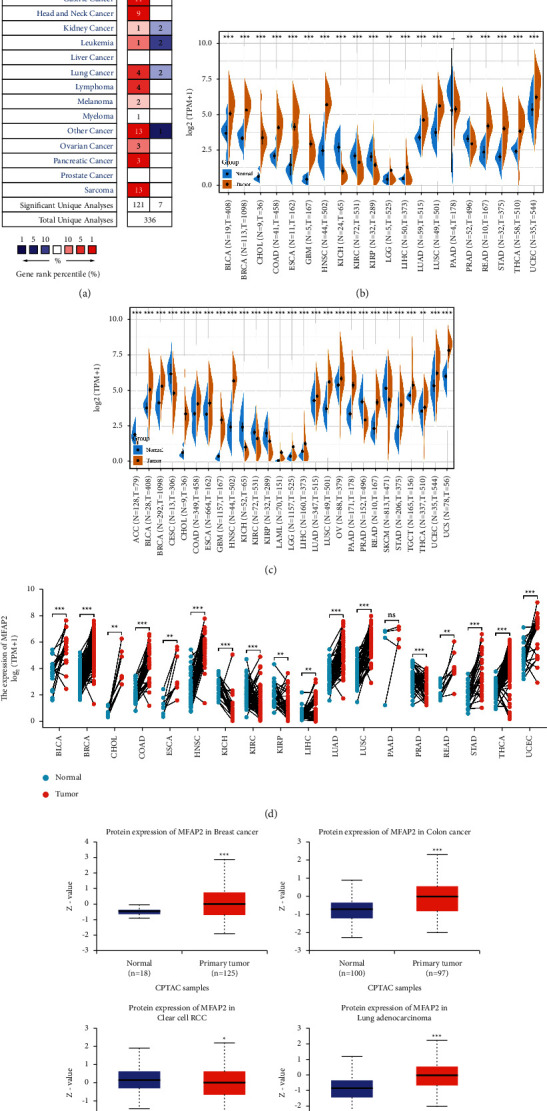
MFAP2 expression landscape in various human cancers. (a) MFAP2 mRNA expression change showed in Oncomine database. (b) MFAP2 mRNA expression levels in tumor tissues and adjacent tissues from TCGA database. (c) MFAP2 mRNA expression levels determined by TCGA database and GTEx database. (d) MFAP2 mRNA expression levels in matched tissues determined by TCGA database. (e) MFAP2 protein expression levels showed in UALCAN database. ^*∗*^*p* < 0.05, ^*∗∗*^*p* < 0.01, and ^*∗∗∗*^*p* < 0.001.

To determine the differential expression of MFAP2, the Oncomine database was used to analyze MFAP2 mRNA levels in different tumors and corresponding normal tissues. This analysis showed that, compared to normal tissues, MFAP2 expression was higher in bladder cancer, brain and central nervous system (CNS) cancer, breast cancer, colorectal cancer, esophageal cancer, gastric cancer, head and neck cancer, lymphoma, melanoma, myeloma, ovarian cancer, pancreatic cancer, and sarcoma. In some datasets of liver cancer and prostate cancer, no difference in expression was observed. In addition, MFAP2 expression was ambiguous in kidney cancer, leukemia, and lung cancer datasets; see Figure 2(a).  Table 2 summarizes the details of MFAP2 expression in various cancers. To further assess the expression of MFAP2 in human cancers, we integrated transcriptomic data from all tumors in TCGA and GTEx. MFAP2 expression obtained from data in TCGA database and integrated data in the GTEx plus TCGA databases are shown in Figure 2(b) and Figure 2(c), respectively. Taking into account individual differences, we further assessed MFAP2 expression in paired samples Figure 2(d). The results showed that MFAP2 was significantly higher in BLCA, BRCA, CHOL, COAD, ESCA, HNSC, LIHC, LUAD, LUSC, READ, STAD, THCA, and UCEC than in adjacent normal tissues and significantly lower in KICH, KIRC, KIRP, and PRAD than in adjacent normal tissues. There was no MFAP2 expression difference in PAAD, which may be due to the small sample size. Moreover, MFAP2 protein expression was elevated in breast cancer, colon cancer, and lung adenocarcinoma and reduced in clear cell renal cell carcinoma; see Figure 2(e).

**Figure 3:**
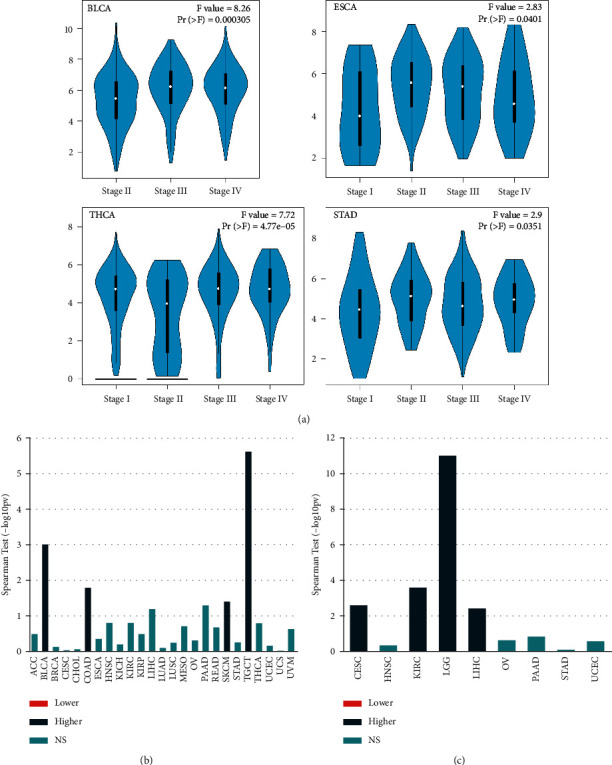
Correlation analysis of MFAP2 expression and clinicopathologic features across various cancers. (a) MFAP2 expression in main pathological stages based on GEPIA2 database. (b) MFAP2 expression in main pathological stages based on TISIDB database. (c) MFAP2 expression in main pathological grades based on TISIDB database.

Based on further analysis of the relationship between MFAP2 mRNA expression levels and cancer stage or grade, the results suggested that MFAP2 was positively correlated with the advanced BLCA stage in both GEPIA2 and TISIDB (see Figures 3(a) and 3(b)), and the TISIDB database of MFAP2 was positively correlated with the advanced CESC, KIRC, LGG, and LIHC grades (see Figure 3(c)).

### 3.2. MFAP2 Prognostic Value across Cancers

**Figure 4:**
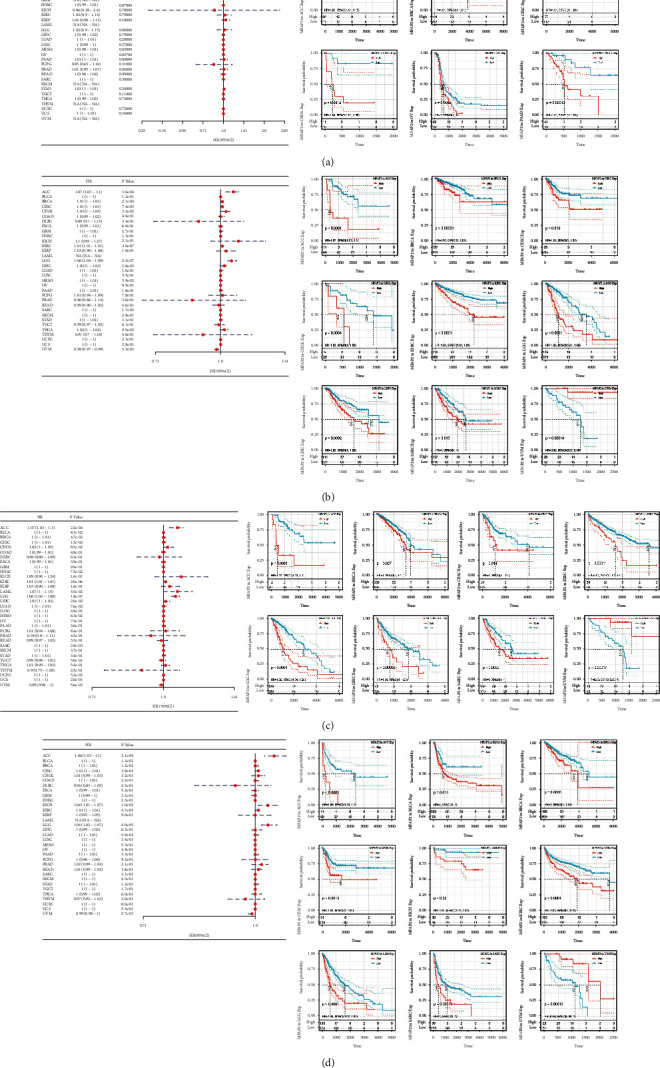
Forest plots and Kaplan–Meier analysis of the association between MFAP2 expression and (a) DFI, (b) DSS, (c) OS, and (d) PFI.

To investigate the relationship between MFAP2 expression levels and prognosis in terms of DFI, DSS, OS, and PFI, we depicted forest plots for each cancer (Figure 4). COX proportional risk model analysis showed that high expression of MFAP2 was associated with poor DFI for ACC, BRCA, CESC, CHOL, OV, and PAAD (Figure 4(a)), with poor DSS for ACC, BRCA, CESC, CHOL, KIRC, LGG, LIHC, and SARC (Figure 4(b)), with poor OS for ACC, BRCA, CESC, KIRC, LGG, LIHC, and SARC (Figure 4(c)), and poor PFI for ACC, BLCA, BRCA, CESC, KICH, KIRC, LGG, and SARC (Figure 4(d)). Interestingly, MFAP2 expression levels in UVM correlated with better DSS, OS, and PFI.

### 3.3. MFAP2 Mutation Profile

**Figure 5:**
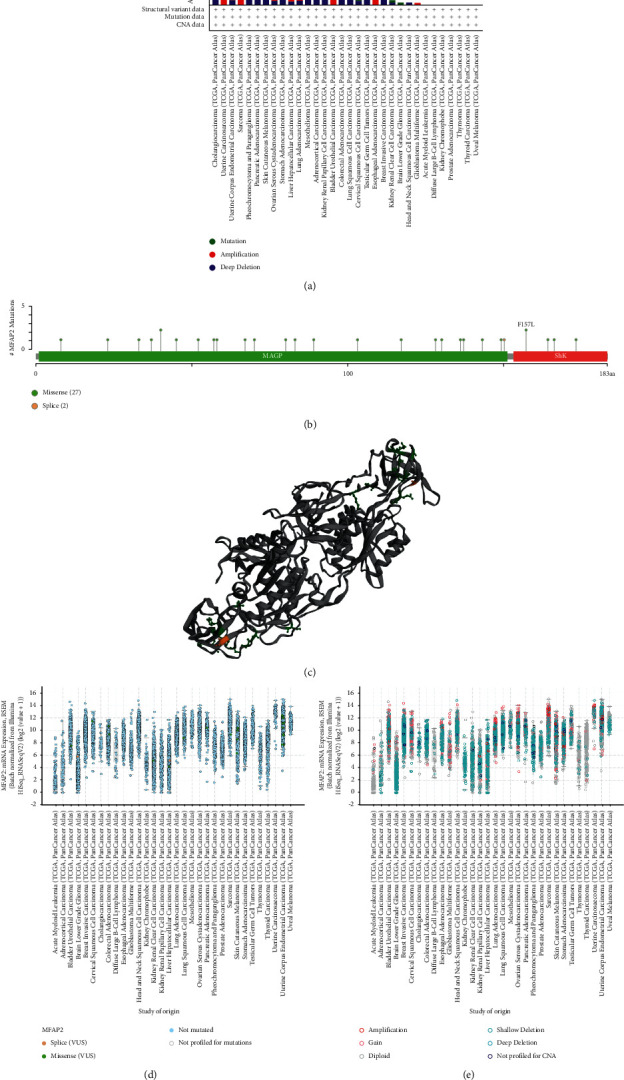
Mutation features of MFAP2 in TCGA pan-cancer panel according to cBioPortal tool. (a) The mutation frequency distribution. (b) The mutation sites distribution across protein domains. (c) The 3D structure of MFAP2. (d) The relevance of mutations and MFAP2 expression. (e) The relevance of DNA copy variation and MFAP2 expression.

Genomic mutations are strongly associated with tumorigenesis. So, we used cBioPortal database to analyze the genomic alterations of MFAP2 in the TCGA pan-cancer datasets, consisting of 10,967 samples from 32 studies. The results showed that cholangiocarcinoma patients with deep deletion as the only mutation type had the highest frequency of MFAP2 alterations, exceeding 5%. In addition, amplification was the only type of MFAP2 mutation in the uterine carcinosarcoma samples, with a mutation frequency of over 3% (Figure 5(a)).  Figure 5(b) shows that between amino acids 0 and 183, a total of 29 MFAP2 mutation sites, including 27 missense and 2 splices, were detected, with missense being the predominant type of DNA alteration. Of these, F157 L in the ShKr protein domain was the most frequent mutant site, detected in 2 cases of endometrial carcinoma. Moreover, the MFAP2 mutant sites were further demonstrated in the 3D structure as shown in Figure 5(c). Additionally, we found that MFAP2 expression was independent of mutations (Figure 5(d)) and independent of DNA copy variation Figure (5(e)).

### 3.4. MFAP2 Aberrant DNA Methylation

**Figure 6:**
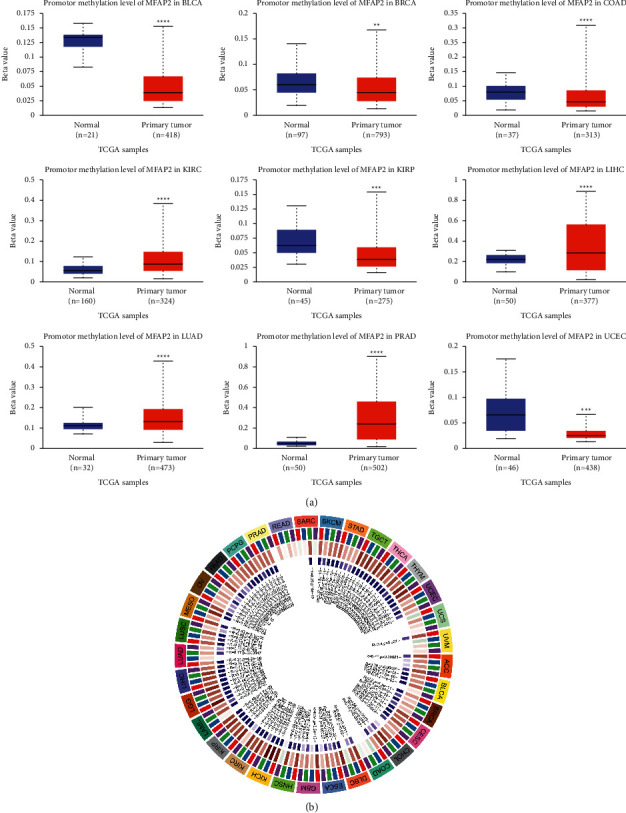
DNA methylation aberration (a) and association with four methyltransferases (b) of MFAP2 in pan-cancer analysis, with DNMT1 in red, DNMT2 in blue, DNMT3A in green, and DNMT3B in purple. ^*∗∗*^*p* < 0.01, ^*∗∗∗*^*p* < 0.001, and ^*∗∗∗∗*^*p* < 0.001.

The genomic alteration analysis suggested that altered MFAP2 expression might not be due to genetic variation. We then examined epigenetic disorders of MFAP2 in cancers. As shown in Figure 6(a), we found that 4 tumors with high MFAP2 expression, including BLCA, BRCA, COAD, and UCEC, exhibited reduced DNA methylation levels of MFAP2. Methylation level of MFAP2 was reduced in KIRP, which could not explain the reduced MFAP2 mRNA expression. In addition, two tumors with low MFAP2 expression, including KIRC and PRAD, exhibited elevated DNA methylation levels of MFAP2. Methylation level of MFAP2 was elevated in LIHC and LUAD, which could not explain the elevated MFAP2 mRNA expression. Then, we assessed the relationship between MFAP2 expression and the four methyltransferases, including DNMT1, DNMT2, DNMT3A, and DNMT3B. As shown in Figure 6(b), MFAP2 expression was positively correlated with some of the four methyltransferases in the vast majority of the 33 tumors.

### 3.5. Tumor Neoantigen, TMB, MSI, and MMRs

**Figure 7:**
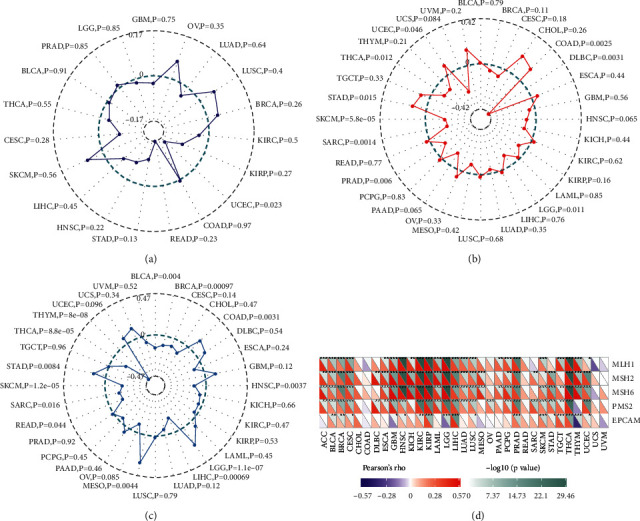
Correlation between MFAP2 expression and the tumor neoantigens (a), TMB (b), MSI (c), and MMRs (d) across cancers. ^*∗*^*p* < 0.05, ^*∗∗*^*p* < 0.01, and ^*∗∗∗*^*p* < 0.001.

Tumor neoantigens are new abnormal proteins encoded by mutated genes in tumor cells, acting as antigens to activate T cells. Here we counted the number of neoantigens for each tumor sample separately and analyzed the relationship between MFAP2 expression and antigens number. The results showed that MFAP2 expression was negatively correlated with tumor neoantigen in UCEC (Figure 7(a)). TMB, usually measured as the number of somatic nonsynonymous mutations occurring in an average of 1 Mb bases in the coding region of the exonic region, reflects the number of mutations contained in tumor cells [18]. Spearman's rank correlation analysis showed that MFAP2 expression was positively correlated with TMB in COAD, STAD, and UCEC, while it was negatively correlated with TMB in DLBC, LGG, PRAD, SARC, SKCM, and THCA as shown in Figure 7(b). MSI refers to the length change of microsatellites due to insertion or deletion of a repeat unit in a tumor compared to normal tissue, producing a new microsatellite allele [19]. Using Spearman rank correlation analysis, MFAP2 expression was positively correlated with TMB in COAD, LGG, MESO, and STAD, while it was negatively correlated with TMB in BLCA, BRCA, HNSC, LIHC, READ, SARC, SKCM, THCA, and THYM (Figure 7(c)). MSI and high TMB may result from MMR deficiency [20]. As shown in Figure 7(d), MFAP2 expression was positively correlated with MLH1, MSH2, MSH6, PMS2, and EPCAM in a variety of tumors, while it was negatively correlated with EPCAM in GBM, LGG, and THYM.

### 3.6. Immune Infiltration Analysis

**Figure 8:**
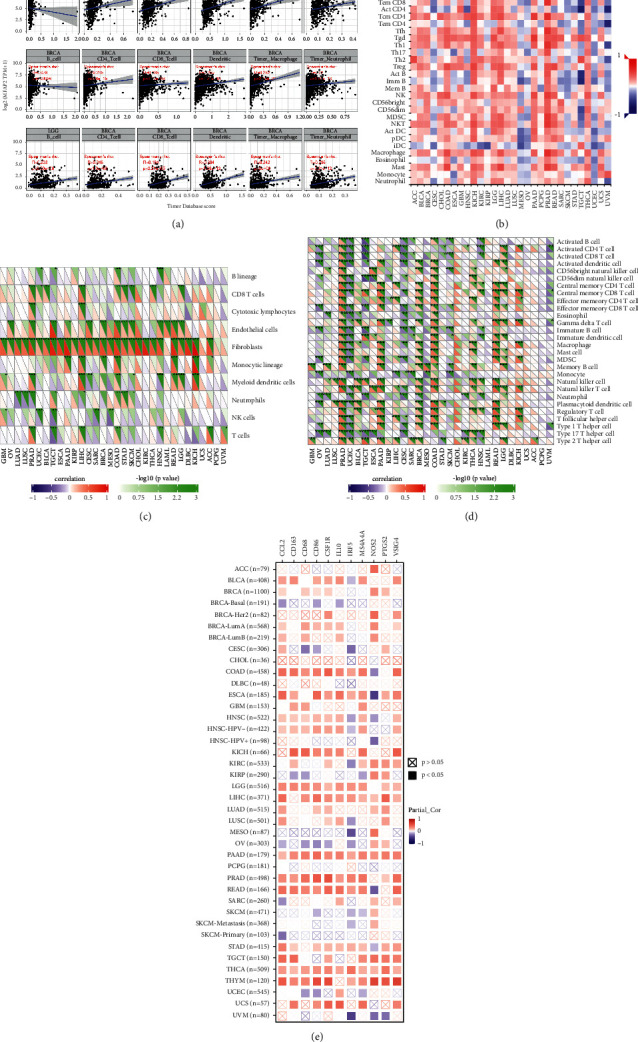
Correlation of MFAP2 expression with immune infiltration in cancers shown in TIMER database (a), TISIDB database (b), MCP analysis (c), and xCell analysis (d). TIMER database showed the correlation of MFAP2 expression with macrophage polarization in cancers, manifested by monocyte markers (CD86 and CSF1R), TAM markers (CCL2, CD68, and IL10), M1 macrophage markers (NOS2, IRF5, and PTGS2), and M2 macrophage markers (CD163, VSIG4, and MS4A4A) (e). ^*∗*^*p* < 0.05, ^*∗∗*^*p* < 0.01, and ^*∗∗∗*^*p* < 0.001.

Tumor-infiltrating lymphocytes, an essential part of the tumor microenvironment, play an essential role in carcinogenesis [21, 22]. Therefore, we analyzed the interaction of MFAP2 with various immune cell infiltration in multiple TCGA cancers by searching TIMER and TISIDB databases or by MCP and XCELL algorithms. In TIMER database, it turned out that, in BLCA, BRCA, and LGG, MFAP2 expression correlated most strongly with the immune infiltration level, with a significant positive correlation with the infiltration levels of B cells, CD4+ T cells, CD8+ T cells, dendritic cells, macrophages, and neutrophils (Figure 8(a)). The detailed correlation in each type of cancer is shown in Supplementary Figure 1. In addition, other algorithms were also performed to assess the correlation. The correlation of MFAP2 expression with immune infiltration in cancers was also analyzed in TISIDB database (Figure 8(b)). MCP analysis (Figure 8(c)) and xCell analysis (Figure 8(d)) were also performed to analyze the correlation, separately. Interestingly, we found that the expression levels of most monocytes, TAMs, M1, and M2 macrophage markers positively correlated with MFAP2 expression levels in BLCA, COAD, ESCA, HNSC-HPV-, KICH, LGG, LIHC, PAAD, PRAD, READ, STAD, THCA, and THYM (Figure 8(e)), suggesting that MFAP2 might regulate macrophage polarization in the aforementioned tumors.

An increasing number of reports have indicated an important role of the tumor immune microenvironment in tumor development [23, 24]. We analyzed the immune and stromal scores of each tumor sample using the *R* package ESTIMATE to observe the relationship between MFAP2 expression in 33 tumors and the StromalScore, ImmuneScore, and ESTIMATEScore (Supplementary Figure 2).

MFAP2 expression in BLCA, BRCA, HNSC, and KICH tissues was shown by qPCR.

**Figure 9:**
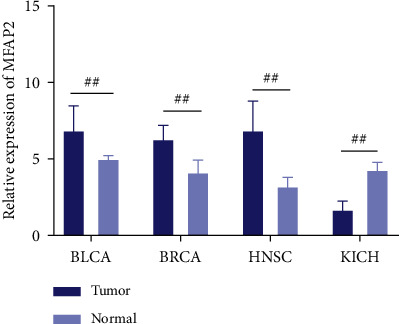
MFAP2 expression was detected in BLCA, BRCA, HNSC, and KICH by qPCR. ^*∗*^*p* < 0.05, ^*∗∗*^*p* < 0.01, ^*∗∗∗*^*p* < 0.001, and ^##^*p* < 0.01.

The expression of MFAP2 was finally examined by qPCR in BLCA, BRCA, HNSC, and KICH tissue samples and the corresponding normal tissue samples. As a result, qPCR showed that MFAP2 expression was significantly lower in KICH tissues than in normal tissues, while it was significantly higher in the other three tumors (Figure 9).

## 4. Discussion

MFAP2 is an essential component of extracellular elastic microfibers, which interacts with and affects fibrin. It is also the constitutive protein of most vertebrate microfibrils [25]. A representative feature of MFAP2 is its capacity to work with TGF-*β* family growth factors, Notch, and Notch ligands, as well as a variety of elastins [26]. Mutations of MFAP2 gene may indicate thrombosis, thoracic aneurysms, metabolic diseases, and osteopenia in humans [27]. Studies have shown that MFAP2 is highly expressed in gastric cancer tissues, and its high expression is significantly related to the overall and disease-free survival of patients with gastric cancer [7]. Furthermore, MFAP2 is found to be a possible player in TGF-*β*/SMAD2/3 signaling pathway activation to advance proliferation, migration, invasion, and epithelial-mesenchymal transition of gastric cancer cells [11]. A previous study pointed out that MFAP2 is a novel microRNA-29 target, and miR-29/MFAP2/integrin*α*5*β*1/FAK/ERK1/2 might be an important carcinogenic pathway in gastric cancer progression [10]. Another study has also indicated the relevance of MFAP2 in hepatic carcinoma, whereby MFAP2 overexpression in hepatic carcinoma is associated with cancer staging, poor OS, and disease-specific survival [9, 26]. An in vitro experiment showed that downregulation of MFAP2 inhibited the proliferation and migration levels of liver cancer cells. Moreover, the transcription factors, DNA methyltransferases, and immune factors in liver cancer might interact with MFAP2 and accelerate tumor progression [27].

In this study, we found that MFAP2 exhibited different expression levels in different tissues and cells. The analysis based on Oncomine, TCGA, GTEx, and UALCAN databases revealed that MFAP2 mRNA and protein were aberrantly expressed in a variety of tumors. However, this aberrant expression was not associated with gene mutations and was influenced to some extent by its promoter methylation. Moreover, the expression of MFAP2 was significantly correlated with the pathological stage, grade, and prognosis of many cancers, suggesting that MFAP2 played an oncogene role in many tumors. In addition, MFAP2 expression was also correlated with DNA methyltransferases, TMB, MSI, and MMR-related genes. Aberrant DNA methylation is frequently seen during cancer progression [28]. MMR is an intracellular MMR mechanism, where the loss of function of key genes may lead to irreparable DNA replication errors and ultimately higher somatic mutations [29]. The correlation between MFAP2 and gene mutations further suggested its importance in tumorigenesis, although such mutations did not produce tumor neoantigens in most cancers.

Combining multiple algorithms, we found that MFAP2 was closely related to the immune infiltration profile in tumor tissue, affecting not only the proportion of immune cells but also the expression levels of many immune-related genes, including immune checkpoints. Among these, a type I transmembrane protein CD276 [30], recently identified as a promising target for tumor immunotherapy [31], is notable. Numerous studies have revealed that CD276 is overexpressed in a variety of tumors, including leukemia [32], breast cancer [33], prostate cancer [34], and other tumors, with expression levels strongly correlating with poor patient prognosis, and presumably involved in tumor immune evasion. In addition, CD276 has been shown to promote lactate production by promoting hexokinase 2 expression, thereby promoting glycolysis and drug resistance [35]. CD276 also could lead to increased NF-*κ*B activity and elevated VEGF expression, further promoting tumor-associated angiogenesis and tumor invasion [36]. Despite unclear underlying mechanisms, the correlation between MFAP2 and CD276 in a variety of tumors suggests that MFAP2 is a promising target for tumor immunotherapy. In the next study, we intend to further verify the effect of MFAP2 on tumor cell proliferation, migration, and invasion and explore the molecular regulation mechanism.

## 5. Conclusion

In summary, our first pan-cancer analysis of MFAP2 suggested that MFAP2 could affect clinical prognosis in various cancers and immune cell infiltration, which deepened the understanding of the MFAP2 role in tumorigenesis.

## Figures and Tables

**Table 1:** PCR primers.

MFAP2	Forward(5′-3′)	CGCCGTGTGTACGTCATTAAC
	Reverse(5′-3′)	CCATCACGCCACATTTGGA
GAPDH	Forward(5′-3′)	TGCCATGTAGACCCCTTGAAG
	Reverse(5′-3′)	ATGGTACATGACAAGGTGCGG

**Table 2:** MFAP2 expression in cancers versus normal tissue in Oncomine database.

Cancer	Cancer type	*P*-value	Fold change	Rank (%)	Sample	Reference (PMID)
Bladder	Infiltrating bladder urothelial carcinoma	1.51E–7	2.327	7%	157	16432078
Bladder	Infiltrating bladder urothelial carcinoma	6.46E–5	3.192	10%	60	15173019
Breast cancer	Invasive ductal breast carcinoma	6.80E–5	4.265	1%	30	17389037
Breast cancer	Invasive ductal breast carcinoma	8.43E–13	3.700	1%	64	15034139
Breast cancer	Lobular breast carcinoma	3.33E–7	3.496	2%	64	15034139
Breast cancer	Ductal breast carcinoma in situ stroma	5.81E–5	2.882	2%	66	19187537
Breast cancer	Tubular breast carcinoma	3.32E–27	2.390	2%	2,136	22522925
Breast cancer	Invasive ductal and invasive lobular breast carcinoma	3.47E–23	2.001	5%	2,136	22522925
Breast cancer	Medullary breast carcinoma	4.21E–8	2.057	8%	2,136	22522925
Breast cancer	Ductal breast carcinoma	6.05E–6	4.753	5%	47	16473279
Colorectal cancer	Colon mucinous adenocarcinoma	4.97E–8	4.413	1%	105	17615082
Colorectal cancer	Cecum adenocarcinoma	3.86E–8	3.135	2%	105	17615082
Colorectal cancer	Rectosigmoid adenocarcinoma	5.22E–6	3.630	2%	105	17615082
Colorectal cancer	Colon adenocarcinoma	2.69E–8	3.485	4%	105	17615082
Colorectal cancer	Colorectal carcinoma	4.42E–12	4.475	1%	105	20957034
Colorectal cancer	Colon carcinoma	3.04E–10	3.539	2%	40	20957034
Colorectal cancer	Colon carcinoma epithelia	5.76E–7	2.270	6%	40	20957034
Colorectal cancer	Rectal adenocarcinoma	1.12E–28	4.306	2%	130	20725992
Esophageal cancer	Esophageal squamous cell carcinoma	7.35E–25	3.246	1%	106	21385931
Esophageal cancer	Esophageal squamous cell carcinoma	3.44E–8	5.112	2%	34	20955586
Gastric cancer	Gastric cancer	5.99E–7	6.107	1%	27	21132402
Gastric cancer	Gastric adenocarcinoma	6.48E–5	2.238	1%	90	21447720
Gastric cancer	Diffuse gastric adenocarcinoma	7.33E–8	2.562	1%	90	21447720
Gastric cancer	Gastric intestinal type adenocarcinoma	6.67E–6	2.656	1%	90	21447720
Gastric cancer	Gastric cancer	1.29E–8	3.563	1%	160	20965966
Gastric cancer	Diffuse gastric adenocarcinoma	1.12E–6	3.264	2%	132	12925757
Gastric cancer	Gastric mixed adenocarcinoma	1.09E–5	4.778	2%	132	12925757
Gastric cancer	Gastric intestinal type adenocarcinoma	4.03E–13	2.947	3%	132	12925757
Gastric cancer	Gastric intestinal type adenocarcinoma	7.03E–12	6.968	2%	69	19081245
Gastric cancer	Gastric mixed adenocarcinoma	1.69E–5	8.780	3%	69	19081245
Head and neck cancer	Head and neck squamous cell carcinoma	1.82E–16	4.913	1%	38	14676830
Head and neck cancer	Salivary gland adenoid cystic carcinoma	2.54E–8	3.510	1%	22	12368205
Head and neck cancer	Head and neck squamous cell carcinoma	8.79E–14	6.381	1%	54	14729608
Head and neck cancer	Oral cavity squamous cell carcinoma epithelia	7.56E–5	2.097	2%	20	15381369
Head and neck cancer	Oral cavity squamous cell carcinoma	1.17E–15	2.621	2%	79	21853135
Head and neck cancer	Tongue carcinoma	2.45E–5	2.754	6%	84	17510386
Lung cancer	Lung adenocarcinoma	2.63E–9	2.443	5%	156	20421987
Lung cancer	Squamous cell lung carcinoma	6.20E–8	2.845	9%	156	20421987
Other cancer	Yolk sac tumor, NOS	3.64E–11	6.521	1%	107	16424014
Other cancer	Mixed germ cell tumor, NOS	4.87E–16	4.189	1%	107	16424014
Other cancer	Teratoma, NOS	5.23E–8	5.705	4%	107	16424014
Other cancer	Uterine corpus leiomyoma	2.49E–10	3.321	1%	77	19622772
Other cancer	Skin basal cell carcinoma	1.65E–7	8.119	1%	87	18442402
Other cancer	Malignant fibrous histiocytoma	1.30E–6	10.342	2%	54	15994966
Other cancer	Pleural malignant mesothelioma	1.63E–5	5.298	3%	54	15920167
Ovarian cancer	Ovarian carcinoma	3.52E–8	3.330	9%	195	18593951
Pancreatic cancer	Pancreatic ductal adenocarcinoma	5.17E–17	4.117	1%	78	19260470
Sarcoma	Dedifferentiated liposarcoma	3.74E–16	6.514	1%	158	20601955
Sarcoma	Myxofibrosarcoma	6.33E–11	4.627	3%	158	20601955
Sarcoma	Pleomorphic liposarcoma	4.94E–8	3.568	3%	158	20601955
Sarcoma	Myxoid/Round cell liposarcoma	4.77E–9	2.956	5%	158	20601955
Sarcoma	Malignant fibrous histiocytoma	1.30E–6	10.342	2%	54	15994966
Sarcoma	Fibrosarcoma	7.42E–6	13.669	2%	54	15994966

## Data Availability

The datasets used and/or analyzed during the current study are available from the corresponding author.

## Supplementary Materials

Supplementary Materials Supplementary Figure 1: correlation of MFAP2 expression with immune infiltration level in diverse kinds of cancers in TIMER database. Supplementary Figure 2: StromalScore (a), ImmuneScore (b), and ESTIMATEScore (c) were analyzed to estimate the correlation of MFAP2 expression with the proportion of immune and stroma in cancers.
