# Supplementary figures and images for: Pan-Cancer Analysis of Microfibrillar-Associated Protein 2 (MFAP2) Based on Bioinformatics and qPCR Verification
Source: J Oncol. 2022 Feb 15;2022:8423173. doi: 10.1155/2022/8423173 (PMC8863482; doi:10.1155/2022/8423173)

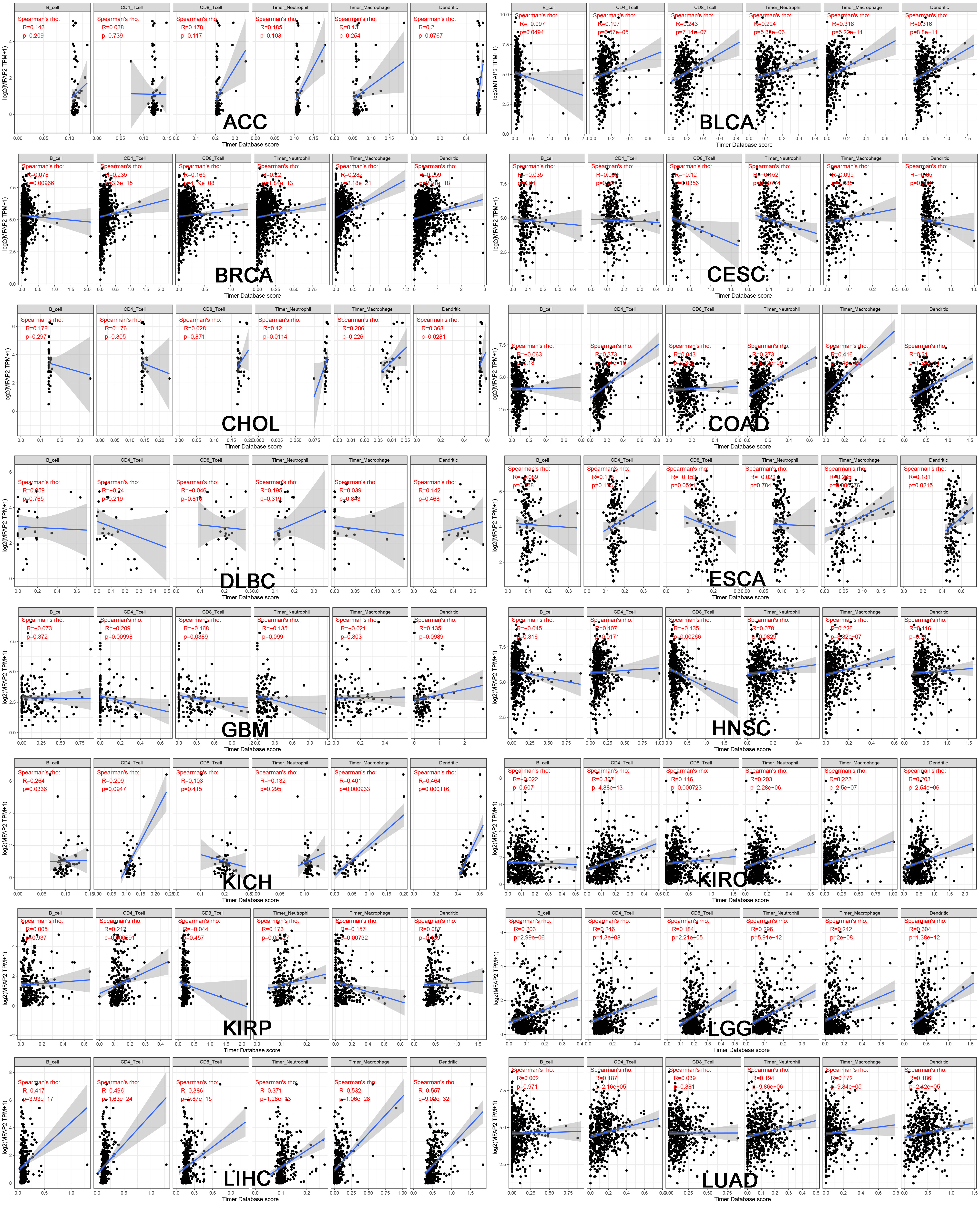

Supplement: Supplementary Materials — Supplementary Figure 1: correlation of MFAP2 expression with immune infiltration level in diverse kinds of cancers in TIMER database. Supplementary Figure 2: StromalScore (a), ImmuneScore (b), and ESTIMATEScore (c) were analyzed to estimate the correlation of MFAP2 expression with the proportion of immune and stroma in cancers. [file 8423173.f1.zip › 8423173.f1/Supplementary Figure 1-1.jpg]

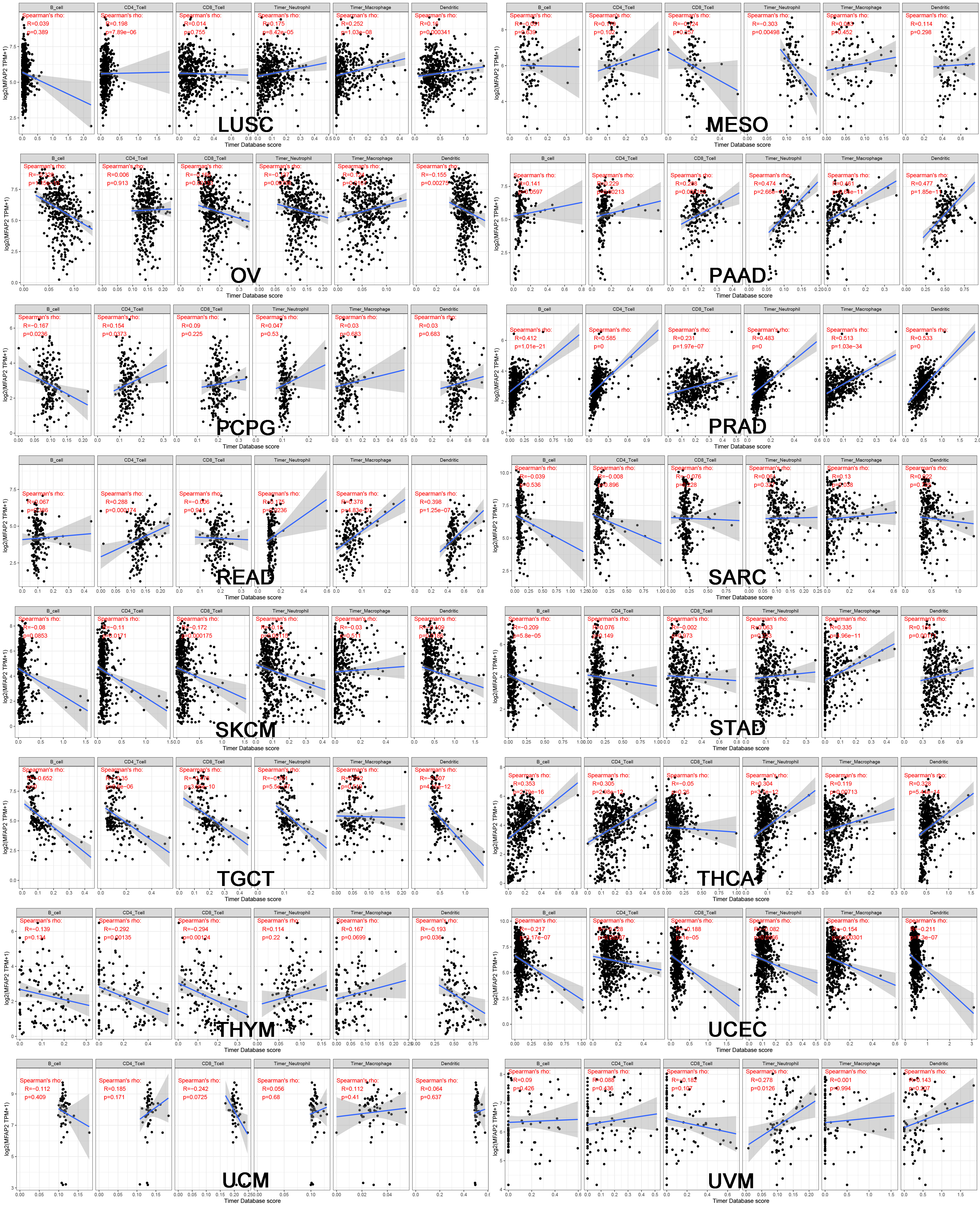

Supplement: Supplementary Materials — Supplementary Figure 1: correlation of MFAP2 expression with immune infiltration level in diverse kinds of cancers in TIMER database. Supplementary Figure 2: StromalScore (a), ImmuneScore (b), and ESTIMATEScore (c) were analyzed to estimate the correlation of MFAP2 expression with the proportion of immune and stroma in cancers. [file 8423173.f1.zip › 8423173.f1/Supplementary Figure 1-2.jpg]

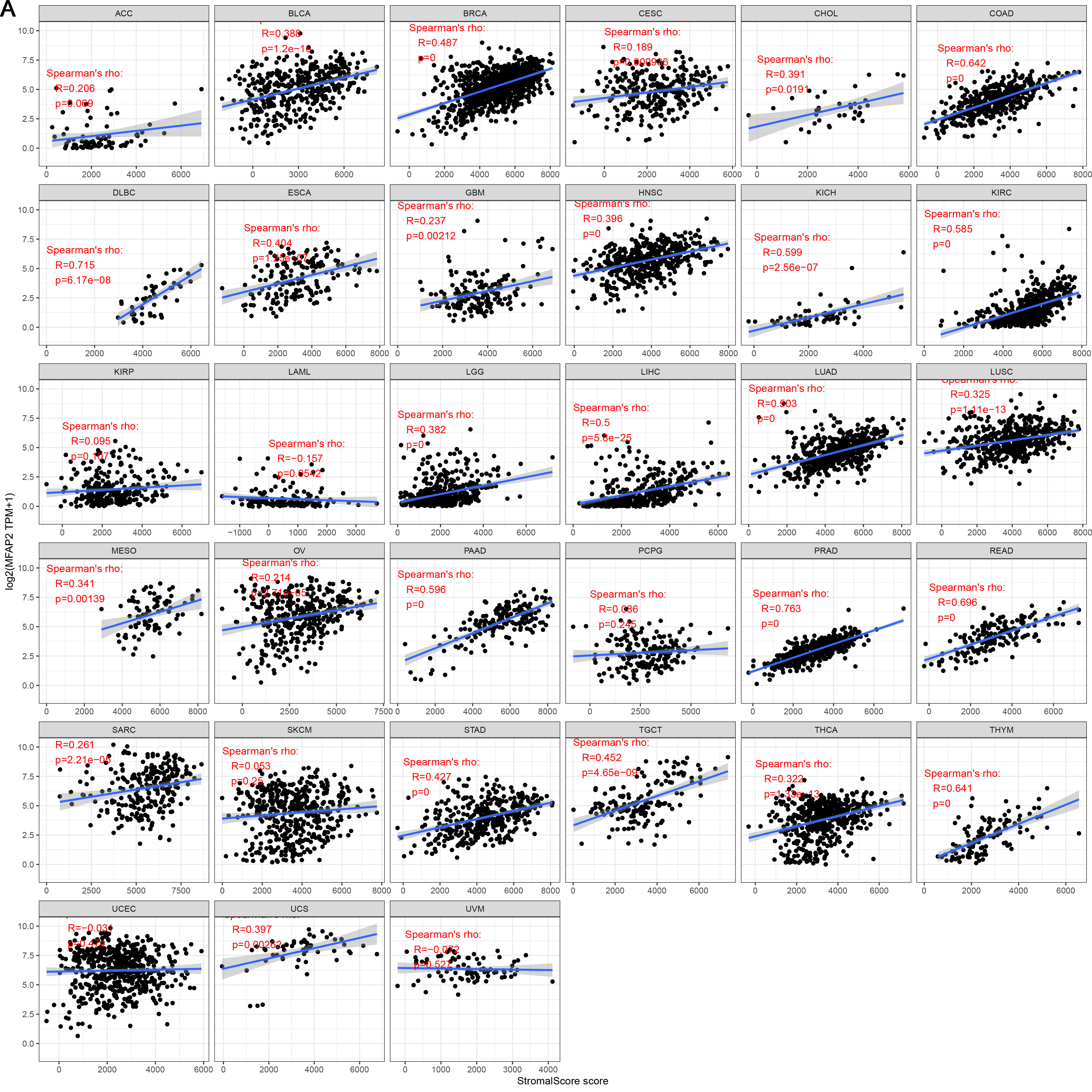

Supplement: Supplementary Materials — Supplementary Figure 1: correlation of MFAP2 expression with immune infiltration level in diverse kinds of cancers in TIMER database. Supplementary Figure 2: StromalScore (a), ImmuneScore (b), and ESTIMATEScore (c) were analyzed to estimate the correlation of MFAP2 expression with the proportion of immune and stroma in cancers. [file 8423173.f1.zip › 8423173.f1/Supplementary Figure 2A.jpg]

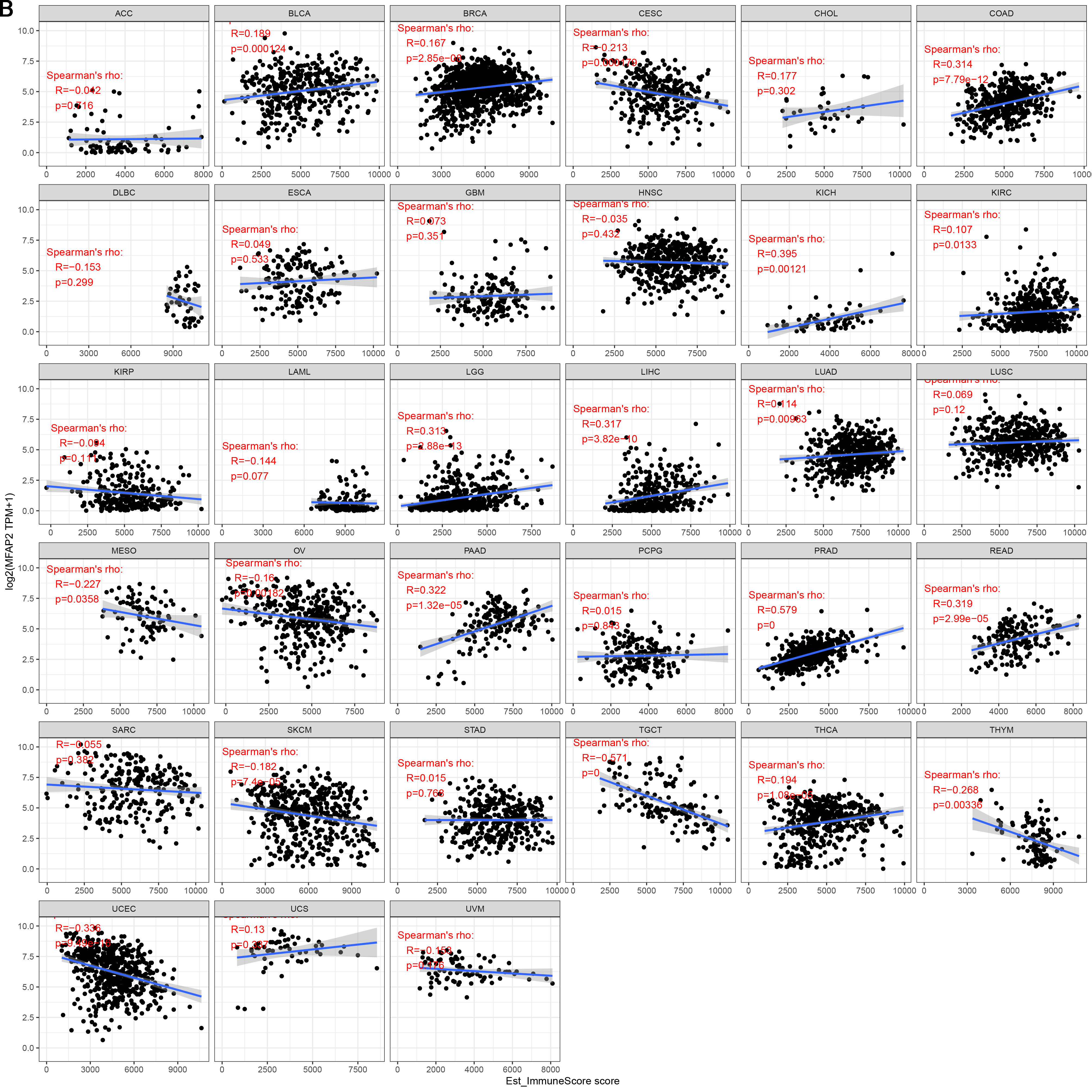

Supplement: Supplementary Materials — Supplementary Figure 1: correlation of MFAP2 expression with immune infiltration level in diverse kinds of cancers in TIMER database. Supplementary Figure 2: StromalScore (a), ImmuneScore (b), and ESTIMATEScore (c) were analyzed to estimate the correlation of MFAP2 expression with the proportion of immune and stroma in cancers. [file 8423173.f1.zip › 8423173.f1/Supplementary Figure 2B.jpg]

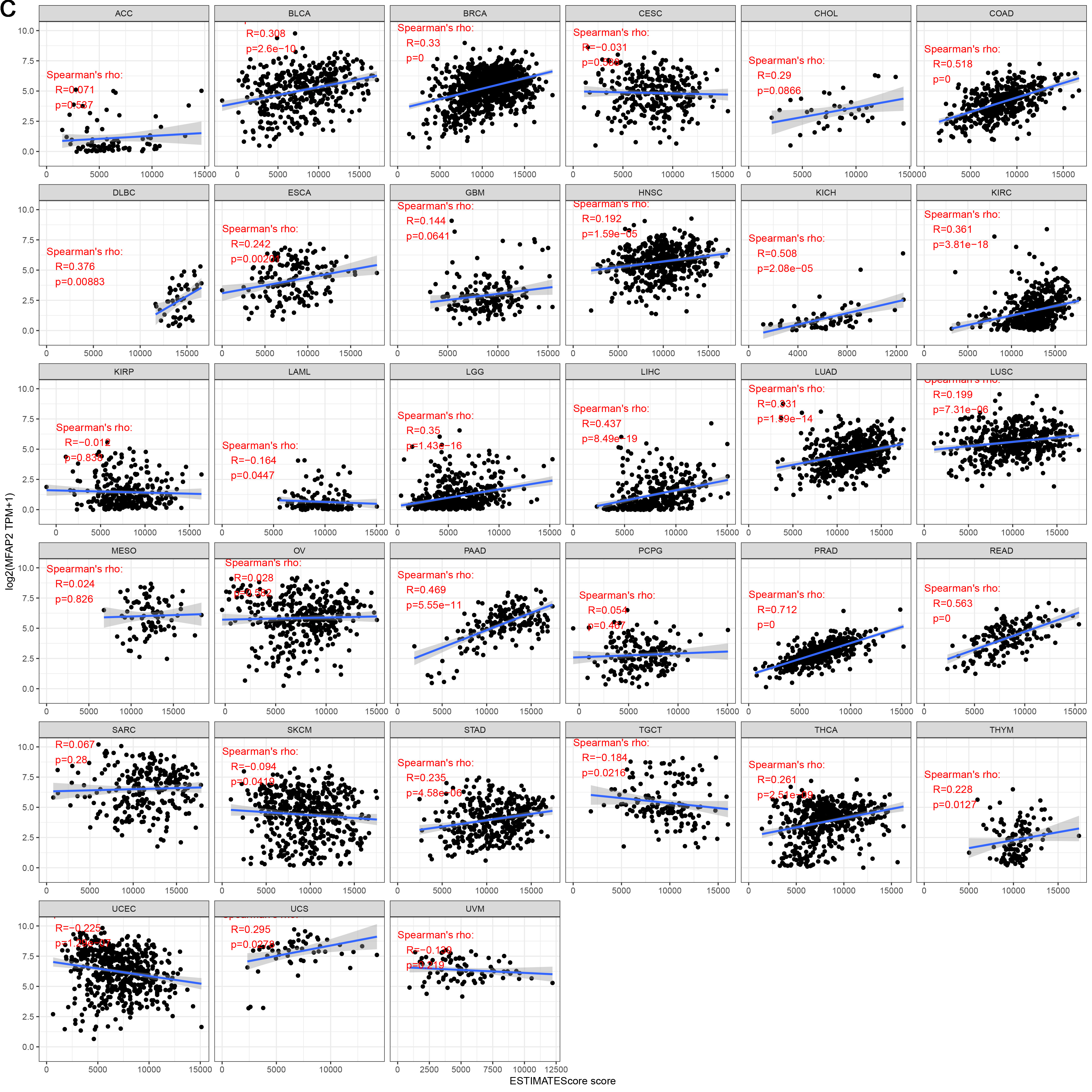

Supplement: Supplementary Materials — Supplementary Figure 1: correlation of MFAP2 expression with immune infiltration level in diverse kinds of cancers in TIMER database. Supplementary Figure 2: StromalScore (a), ImmuneScore (b), and ESTIMATEScore (c) were analyzed to estimate the correlation of MFAP2 expression with the proportion of immune and stroma in cancers. [file 8423173.f1.zip › 8423173.f1/Supplementary Figure 2C.jpg]
